# MPSSS impairs the immunosuppressive function of cancer-associated fibroblasts via the TLR4-NF-κB pathway

**DOI:** 10.1042/BSR20182171

**Published:** 2019-05-10

**Authors:** Yuwei Xu, Jing Ma, Qilin Zheng, Yuanyuan Wang, Minghua Hu, Fangli Ma, Zhihai Qin, Ningjing Lei, Ning Tao

**Affiliations:** 1 Medical Research Center, The First Affiliated Hospital of Zhengzhou University, Zhengzhou University, Zhengzhou 450052, China; 2 Key Laboratory of Protein and Peptide Pharmaceuticals, Institute of Biophysics, Chinese Academy of Sciences, Beijing 100101, China; 3 College of Life Science, University of Chinese Academy of Sciences, Beijing 100101, China; 4 School of Basic Medical Sciences of Southwest Medical University, Luzhou 646000, China; 5 Infinitus Chinese Herbal Immunity Research Centre, Infinitus Company Ltd, Guangzhou 510635, China

**Keywords:** antitumor immunity, cancer-associated fibroblasts, immunosuppression, Lentinus edodes polysaccharides, prostate cancer

## Abstract

The polysaccharides MPSSS was extracted from *Lentinus edodes* and has been reported to effectively inhibit tumor growth and eliminate the function of myeloid-derived immune suppressor cell-mediated T cell inhibition, thus improving the efficacy of cancer therapy. The exploration of how MPSSS affects the functions of cancer-associated fibroblasts (CAFs) will provide a new perspective for understanding the antitumor effects of MPSSS. In the present study, prostate CAFs were selected as target cells to study whether MPSSS affected cell proliferation and function. The results showed that MPSSS did not directly inhibit the growth of prostate CAFs but interfered with CAF-mediated T cell inhibition and affected the immunosuppressive function of prostate CAFs. Mechanistic studies were further performed and showed that MPSSS activated key node proteins in the NF-κB pathway that were dependent on MyD88, and a TLR4 inhibitor blocked the changes in these proteins and the effect of MPSSS. We hypothesize that MPSSS can activate the MyD88-dependent TLR4-NF-κB signaling pathway to change the function of CAFs. In conclusion, these results demonstrate that MPSSS can not only effectively inhibit the growth of prostate cancer as we previously reported but also alter the function of prostate CAFs by activating the TLR4-NF-κB pathway, providing a new strategy for the comprehensive treatment of tumors.

## Introduction

Prostate cancer is a cutaneous malignancy that occurs in the prostate and may spread from the prostate to other areas of the body, especially to the bones and lymph nodes [1]. In recent years, the incidence of prostate cancer in China has been increasing and has become the second most common malignancy in males following skin cancer. Therefore, the treatment of prostate cancer is currently an important topic in biomedical engineering [2]. For a long time, the occurrence and progression of cancer have been attributed to malignant cells that have ability to proliferate, invade, and metastasize. However, studies have shown that tumor progression is not entirely dependent on the accumulation of cancer cells with gene mutations and the tumor microenvironment also plays an important role [3]. Fibroblasts, endothelial cells, and immune cells are the main cell types in the tumor microenvironment and are usually immunosuppressive; tumor cells can escape the body’s immune defense and then can rapidly grow and spread. As one of the main components in the tumor microenvironment, the matrix encapsulates tumor cells and forms a network of connective tissues around the tumor.

Cancer-associated fibroblasts (CAFs) are one of the major types of stromal cells. Compared with the changes to normal-associated fibroblasts (NAFs) in tissues, CAFs show significant phenotypic and functional changes and are activated [4]. In the tumor microenvironment, an increase in transforming growth factor (TGF) levels can induce NAFs to undergo a phenotypic change to CAFs and to express specific proteins, such as α*-*smooth muscle actin (α-SMA) and fibroblast activation protein (FAP) [5]. Once CAFs appear in the tumor microenvironment, they will change the composition and biochemical characteristics of the extracellular matrix and will promote the growth and dissemination of tumor cells. Stromal fibroblasts, located at the interface between tumor endothelial cells and adjacent tissues, interact with tumor, endothelial, and immune cells in hypoxic environments; CAFs affect the tumor immune response by secreting cytokines, chemokines, and angiogenic factors. For example, IL-6 signals that are derived from CAFs restrict the maturation of dendritic cells and the activation of T cells that can induce T cell anergy. In addition, CXCL9, CXCL10, and stromal-derived factor-1 (SDF-1) that are secreted by CAFs inhibit the recruitment of T cells, attract inhibitory T cells, and lead to the uncontrolled activity of effector T cells, which interferes with the interactions between T and tumor cells, and finally prevents the destruction of tumor [6]. In addition to acting directly on the immune system, CAFs can indirectly regulate tumor immunity by influencing tumor angiogenesis. The removal of FAP^+^ fibroblasts does not alter the number or type of tumor-infiltrating T cells but can activate the cells and the secretion of inflammatory factors, such as interferon-γ (IFN-γ) and tumor necrosis factor-α (TNF-α), which leads to anoxic necrosis of cancer and stromal cells promoting the immune-mediated control of tumor growth. In turn, the inhibition of fibroblasts can suppress tumor growth via CAF-targeted immunotherapy [7].

As immunomodulators and antitumor agents, polysaccharides can indirectly inhibit or kill tumor cells by improving host immune functions or by directly playing an antitumor role by inducing the differentiation or apoptosis of tumor cells and by affecting oncogene expression [8]. In addition, polysaccharides have relatively few side effects on the human body. At present, lentinan, a polysaccharide extracted from *Lentinus edodes*, has been used as a supplementary drug in cancer treatment [9,10]. MPSSS is a new polysaccharide isolated from *L. edodes*. Its molecular weight is 577.2 kDa, and it is mainly composed of glucose (75.0%), galactose (11.7%), mannose (7.8%), and xylose (0.4%). In previous studies, we found that MPSSS could induce the differentiation of myeloid-derived suppressor cells (MDSCs) and could decrease their immunosuppressive functions [11]. Nevertheless, whether MPSSS can improve the tumor microenvironment and whether it has an effect on CAFs have not been studied. In addition, the underlying mechanism of MPSSS has not been determined. The present study explored the effects of MPSSS on CAF functions and the associated mechanism, which may provide new antitumor strategies.

## Materials and methods

### Purification of MPSSS

Crude polysaccharides from *L. edodes* were supplied by the Infinitus Chinese Herbal Immunity Research Centre in Guangzhou, China. Crude polysaccharides from *L. edodes* were dissolved overnight in de-ionized water and were then centrifuged to obtain the supernatant. Anhydrous ethanol was added to the supernatant at a 1:1 volume and was stored in a refrigerator at 4°C overnight. After centrifugation, the precipitate was collected and dissolved in a 30% (v/v) ethanol solution. Anhydrous ethanol was added to the supernatant after centrifugation to form a 40% (v/v) ethanol solution. After 12 h, the solution was similarly processed to obtain a 50% (v/v) ethanol solution. When the concentration of ethanol reached 80% (v/v), the precipitate was collected. Finally, MPSSS was obtained as a grey powder after washing and drying the precipitate with anhydrous ethanol and anhydrous ether. For use, 10 mg/ml MPSSS solution was obtained by adding a certain amount of DMEM to fully dissolve the MPSSS powder.

### Preparation of CAF and NAF supernatants

NAFs and prostate CAFs were cultured in DMEM (HyClone, U.S.A.) supplemented with 10% FBS (PAN, Germany) and 1% penicillin/streptomycin at 37°C and 5% CO_2_ in an incubator with a humidified atmosphere. NAFs and CAFs were seeded into 48-well plates at a density of 2 × 10^5^ cells/well and were cultured in 300 μl DMEM medium. Then, different concentrations of MPSSS solution (0, 0.1, 0.2, 0.3, 0.4, and 0.5 mg/ml; six replicate wells for each concentration) were added to the wells, and the cells were incubated for 24 or 48 h. Then, the medium was removed from the wells containing MPSSS, and new DMEM was added. After 24 h, the medium from each well was collected and filtered with a 0.22 μm filter to obtain the supernatant. The supernatant used for the TLR4 inhibition assay was derived from cells that were previously treated with 1 μg/ml CIL-095 for 6 h and were then incubated with MPSSS.

### Cell viability assay: 3-[4,5-dimethylthiazol-2-yl]-2,5-diphenyltetrazolium bromide (MTT) assay

A 3-[4,5-dimethylthiazol-2-yl]-2,5-diphenyltetrazolium bromide (MTT) assay was used to evaluate the MPSSS-induced cytotoxicity. Prostate CAFs were seeded into 96-well flat plates at a density of 5000 cells/well and were incubated with MPSSS at different concentrations for 24 or 48 h. Then 10 µl of the MTT reagent was added to each well and was incubated for 4 h. After dissolving the bluish-violet crystals with a three-solution mixture (10% SDS, 5% isobutanol, 0.012 mol/l HCl, dissolved in distilled water) for 4–6 h, the absorption at 570 nm was measured with a microplate reader (BIO-RAD Laboratories, Philadelphia, PA, U.S.A.). For experiments to detect the effects of MPSSS on splenocyte proliferation, splenocytes were obtained from wild-type mice and were cultured in 96-well U-bottom plates at a cell density of 3 × 10^5^ cells/well, using concanavalin A (ConA) as the positive control and DMEM+/+ without MPSSS and MPSSS-untreated CAF supernatants as the negative controls. In the experimental group, MPSSS at different concentrations or CAF supernatants pretreated with different doses of MPSSS were added to stimulate the splenocytes for 24 h, and then splenocyte viability was measured with the same process described above.

### T cell proliferation: flow cytometry

T cell proliferation was measured using the intracellular dye carboxyfluorescein succinimidyl amino ester (CFSE). Splenocytes were isolated from wild-type BALB/c mice and were incubated in RPMI-1640 medium for 4 h. The splenocytes were centrifuged and collected, and then cell suspensions with concentrations of 1 × 10^7^ cells/ml were labeled with 2 μm/ml CFSE at 37°C for 5 min, followed by quenching in 10% FBS-RPMI 1640 medium. CFSE-labeled splenocytes were co-cultured at a density of 3 × 10^5^ cells/well and were treated with 0.6 µg/ml ConA and MPSSS at different concentrations. For the prostate-CAFs and splenocytes co-culture system, the CAFs were pretreated with 0.5 mg/ml MPSSS for 24 h, and then the CFSE-labeled splenocytes at a density of 3 × 10^5^ cells/well were co-incubated with 100 CAFs/well in 96-well U-bottom plates. Negative controls (with 0.6 μg/ml ConA and 30% MPSSS-untreated CAF supernatant or untreated prostate CAFs) and a positive control (0.6 μg/ml ConA) were set up in the experiment. After 3 days, the cells were stained with APC-labeled anti-CD4 and PE-labeled anti-CD8a antibodies, and the proliferation rates of CD4^+^ and CD8^+^ cells among the CFSE^+^ cells were detected by FACS. The antibodies, including APC-labeled anti-CD8a and PE-labeled anti-CD4, were diluted in PBS with 3% FBS.

### Western blot assay

Prostate CAFs were seeded in 6-well plates at a density of 3 × 10^5^ cells/well and were treated with MPSSS at different concentrations (0, 0.1, 0.2, 0.3, 0.4, and 0.5 mg/ml) for 24 h. Then the cells were lysed with RIPA buffer supplemented with 100 mΜ phenylmethylsulfonyl fluoride (PMSF), 25 μg/ml aprotinin, 1 mM sodium orthovanadate, and 50 nM NaF to obtain the total protein content. The total protein concentrations were determined by the BCA protein assay. Equal amounts of protein samples (30 μg/sample) were separated on 10% SDS polyacrylamide gels under denaturing conditions and were then electrotransferred onto nitrocellulose membranes (GE Healthcare, Milwaukee, WI, U.S.A.) for 70 min at 100 V. Then, the membranes were blocked with 3% BSA in PBS-T (0.1% Tween-20) for 1 h and were incubated overnight at 4°C with the primary antibodies. The primary antibodies used were against α-SMA, phospho-NF-ĸB p65, NF-ĸB p65, phospho-TAK, TAK, phospho-IKKα/β, TRAF6, and MyD88 (all from Cell Signaling Technology, U.S.A.) and were diluted at 1:1000; the primary antibody against GAPDH (Tianjin Sungene Biotech Co., Ltd.) was diluted at 1:2000 and was used as an internal standard.

## Results

### MPSSS exerts no direct effect on the viability of prostate CAFs

**Figure 1:**
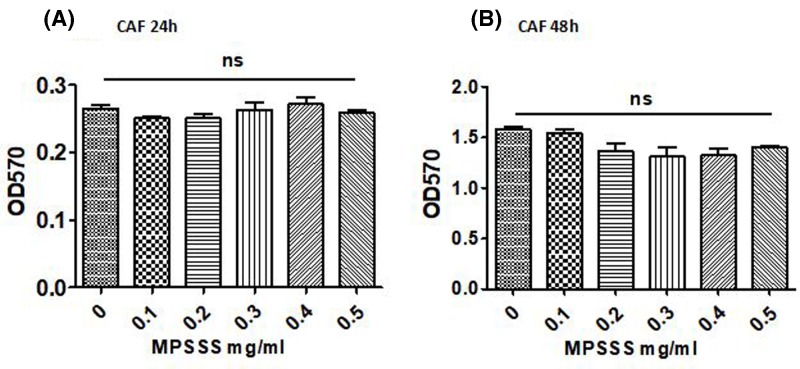
MPSSS exerts no toxicity in prostate CAFs Prostate CAFs (5000 cells/well) were treated with MPSSS at different concentrations for 24 h (**A**) and 48 h (**B**). Cell viability was measured at OD570. ns represents no statistically significant difference; *** *P*<0.001.

MTT assays were used to test the direct effect of MPSSS on prostate CAFs. Prostate CAFs were treated with MPSSS at different concentrations (0–0.5 mg/ml). The results showed that there were no significant differences in the viability of prostate CAFs after treatment with MPSSS for 24 h (Figure 1A). When the incubation time was prolonged to 48 h, there were still no significant differences in the cell activity (Figure 1B), indicating that MPSSS exerts no toxicity in prostate CAFs.

### MPSSS does not affect the activity of splenocytes but reduces the inhibitory effect of prostate CAFs on splenocytes

**Figure 2:**
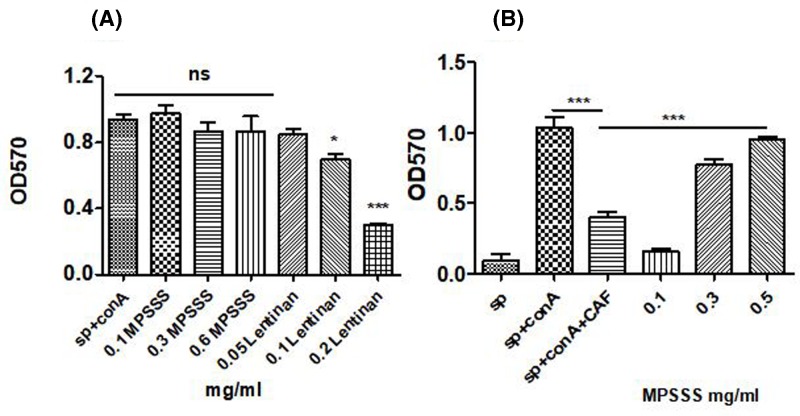
MPSSS impairs the inhibitory effect of prostate CAFs on splenocytes without influencing the activity of splenocytes (**A**) Upon ConA treatment, changes in splenocyte proliferation were measured after treatment with lentinan and MPSSS at different concentrations. (**B**) Splenocytes were incubated with 30% MPSSS-treated or MPSSS-untreated prostate CAF supernatant, and then the changes in the proliferation rates were evaluated. ** *P*<0.01, * *P*<0.1.

To detect the effect of MPSSS on splenocytes, we used MTT assays to determine whether MPSSS could directly promote the growth of splenocytes. Lentinan is used in clinical practice and was used here as a control. The results showed that the number of splenocytes incubated with MPSSS at different concentrations for 24 h was not significantly different from the number of untreated cells (Figure 2A). However, lentinan inhibited the proliferation of splenocytes, and the inhibitory effect was enhanced with increasing concentrations of lentinan. MPSSS had no direct effect on the proliferation of splenocytes; however, when cultured with 30% MPSSS-treated CAF supernatants, the inhibitory effect of prostate CAFs on the proliferation of splenocytes decreased, and a dose-dependent relationship was observed (Figure 2B).

### MPSSS impairs the immunosuppressive function of prostate CAFs

**Figure 3:**
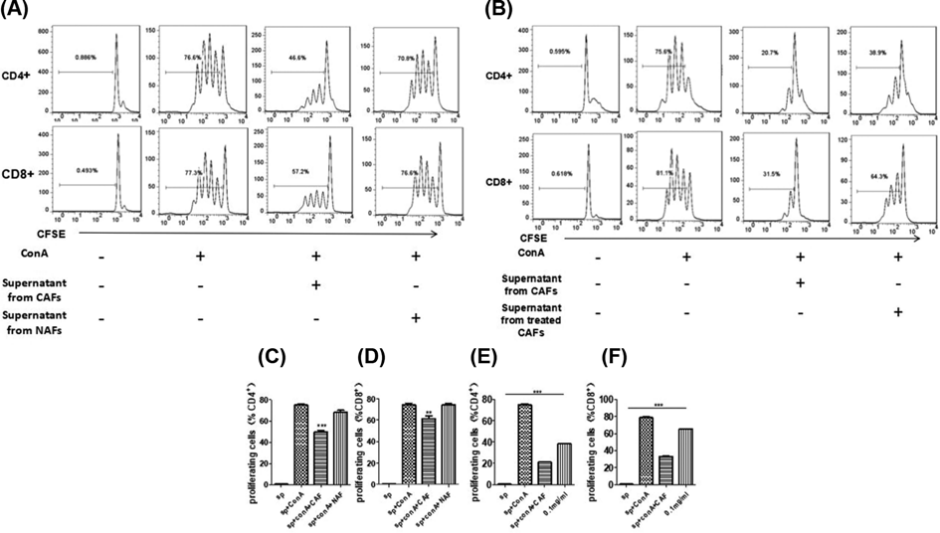
Effect of MPSSS on the immunosuppressive function of prostate CAFs (**A**) CFSE-labeled splenocytes were cultured with NAF supernatant or prostate CAF supernatant and were treated with or without ConA. The CD4^+^ and CD8^+^ cell proliferation rates were assessed by flow cytometry after 3 days. (**B**) CFSE-labeled splenocytes were cultured with untreated or MPSSS-treated CAF supernatant. The quantification of (**C,E**) CD4+ or (**D,F**) CD8^+^ cell proliferation was expressed as the mean ± SD from triplicate determinations and was analyzed by one-way ANOVA.

To further verify the effect of MPSSS on the immunosuppressive function of prostate CAFs, we obtained splenocytes from wild-type BALB/c mice; we then stained them with the fluorescent dye CFSE and incubated them with 0.6 μg/ml ConA. The cells were suspended at a density of 3 × 10^5^ cells/well and were cultured in 30% prostate CAF supernatant until treatment with 0.1 mg/ml MPSSS for 48 h. Meanwhile, NAF supernatant was used as a control. After 3 days, the proliferation of CD4^+^ and CD8^+^ T cells was measured by flow cytometry. The results showed that the rate of T cell proliferation decreased after the addition of the supernatant from prostate CAFs, but the supernatant of NAF had no obvious inhibitory effect on T cell proliferation (Figure 3A). More importantly, treatment with MPSSS attenuated the inhibitory effect of CAF on T cell proliferation (Figure 3B). A statistical analysis of the rate of T cell proliferation showed that prostate CAFs could inhibit the proliferation of CD4^+^ (Figure 3C,E) and CD8^+^ (Figure 3D,F) T cells *in vitro*. However, MPSSS attenuated the inhibitory effect of CAFs.

### MPSSS weakens the immunosuppressive function of prostate CAFs in a co-culture system

**Figure 4:**
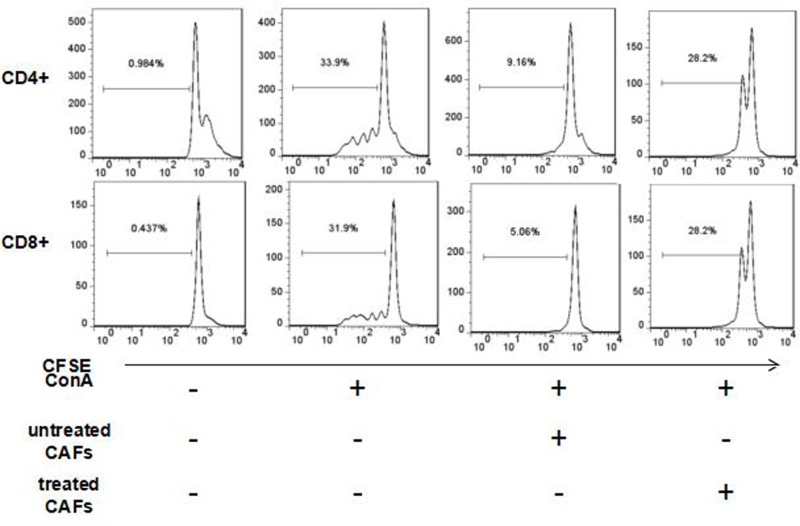
MPSSS weakens the immunosuppressive function of prostate CAFs in a co-culture system Activated CFSE-labeled splenocytes were co-cultured with pretreated prostate CAFs and were incubated with ConA, and the proliferation of CD4^+^ and CD8^+^ T cells was assessed by flow cytometry.

A supernatant culture system was used, and the results showed that MPSSS could inhibit the proliferation of T cells by changing the function of CAFs and by influencing the levels of immunosuppressive factors in the supernatant; however, the possibility that CAFs directly interacting with the T cells could not be ruled out. To verify this possibility, we designed a co-culture system. Prostate CAFs were pretreated with or without 0.5 mg/ml MPSSS for 24 h and were co-cultured with CFSE-labeled splenocytes at a ratio of 1:3000 in a 96-well round plate, and 0.6 µg/ml ConA was added to the cells at the same time. The proliferation rate of CD4^+^ and CD8^+^ T cells among the splenocytes was measured after culturing the cells at 37°C for 3 days. The results showed that MPSSS-untreated CAFs significantly decreased the proliferation of T cells, but the inhibitory effect of CAFs on T cell proliferation was significantly weakened after incubation with MPSSS (Figure 4). These results suggest that prostate CAFs may directly interact with T cells, thus affecting their proliferation. MPSSS can also impair the inhibitory effects of CAFs on T cells. However, whether CAFs regulate the immune microenvironment by interacting with T cells directly and how they interact with each other need further verification.

### MPSSS changes the function of prostate CAFs via the TLR4-NF-κB pathway

**Figure 5:**
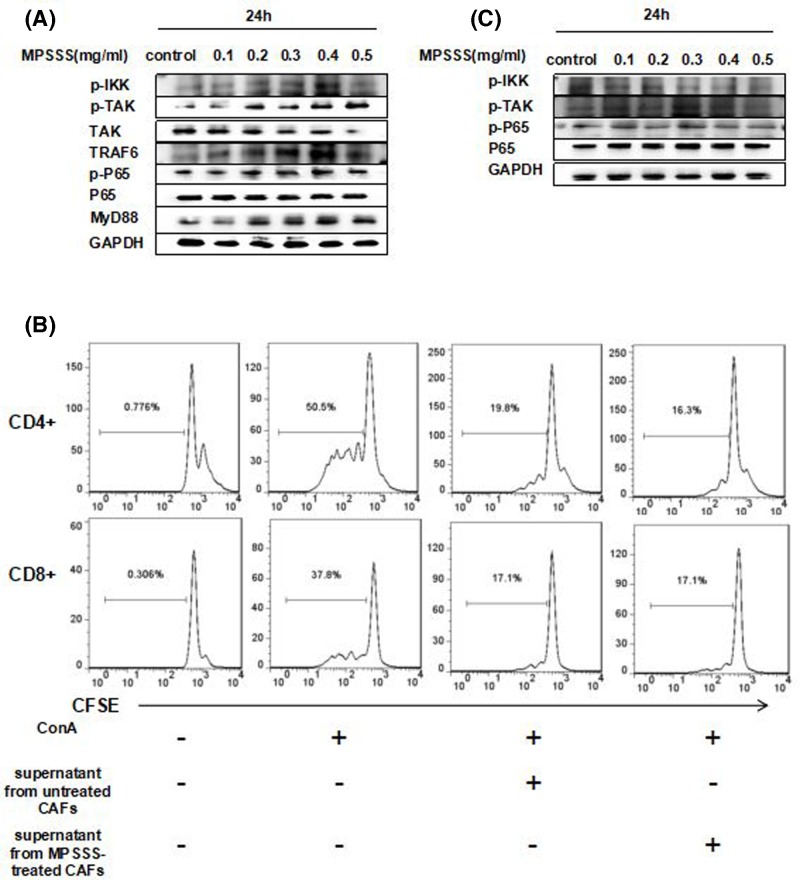
MPSSS changes the function of prostate CAFs via the TLR4-NF-κB pathway (**A**) After incubation with different concentrations of MPSSS as indicated, the total protein content was collected, and the levels of p-IKKα/β, p-TAK, TAK, TRAF6, p-p65, p65, MyD88, and GAPDH were analyzed by Western blot assays. (**B**) Prostate CAFs were pretreated with 1 µg/ml of the TLR4 inhibitor CIL-095 and were then incubated with 0.1 mg/ml MPSSS for 48 h. A 30% dilution of the supernatant from treated CAFs was used to culture splenocytes, and the rate of T cell proliferation was measured. (**C**) After pretreatment with 1 µg/ml CIL-095, prostate CAFs were incubated with MPSSS at different concentrations, and the expression levels of the proteins related to the NF-κB pathway were detected.

To investigate the mechanism of MPSSS in CAFs, we used Western blot assays to identify the possible pathways affected by MPSSS. Our previous studies on MPSSS have shown that MPSSS could induce p65 phosphorylation in MDSCs and that MPSSS plays a role through the MyD88-dependent NF-ĸB pathway [11]; therefore, we speculated that MPSSS affect prostate CAFs via a similar mechanism. After incubation with different concentrations of MPSSS (0–0.5 mg/ml) for 24 h, Western blot results showed that the expression of proteins related to the activation of the MyD88-dependent NF-ĸB pathway was significantly up-regulated, suggesting that MPSSS could activate the NF-ĸB pathway in prostate CAFs and could change their functions (Figure 5A). Moreover, it has been reported that MyD88 often interacts with Toll-like receptors and causes changes in the downstream pathway [12]. In addition, among all Toll-like receptors, TLR4 binds to lipopolysaccharides (LPS) [13,14]. Since both MPSSS and LPS are polysaccharides, it can be assumed that TLR4 may be the receptor for MPSSS. To verify this hypothesis, we used the TLR4 inhibitor CIL-095 to block TLR4 and detected the effects of MPSSS on prostate CAFs. The flow cytometry results showed that the inhibitory effect of CIL-095-pretreated CAFs on T cell proliferation could not be decreased by MPSSS (Figure 5B). Western blot confirmed these results. After co-treatment with receptor blockers and MPSSS, the expression of proteins related to NF-ĸB pathway activation in CAFs did not significantly change (Figure 5C). Therefore, we proved that MPSSS changes the function of prostate CAFs through the TLR4-NF-ĸB pathway.

### MPSSS reduces the activity of prostate CAFs

**Figure 6:**
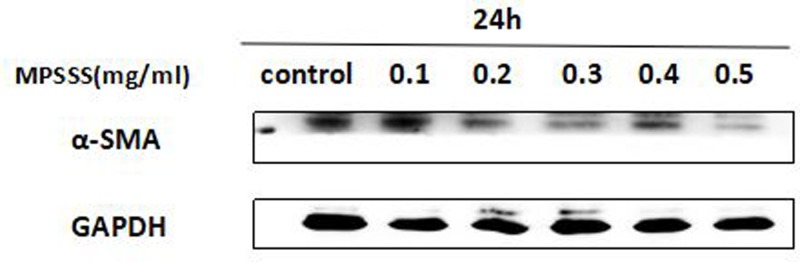
MPSSS reduces the activity of prostate CAFs After treatment with MPSSS, the expression of α-SMA as a marker of CAF activity was analyzed by Western blotting. GAPDH served as a control.

Under normal conditions, fibroblasts maintain tissue homeostasis and contribute to proper cell communication and function. In malignant tumor environments, a set of factors secreted from cancer or immune cells could activate fibroblasts. Once activated, fibroblasts undergo a phenotype switch and become CAFs, which express various markers such as α-SMA, FAP, vimentin, and periostatin [15]. Fibroblasts with high expression levels of these markers are often located at the junction of tumor endothelial cells and the surrounding tissues, and they block the normal response of the immune system to tumors and inhibit the interaction between T cells and tumor cells, thus preventing tumor destruction [4]. To further elucidate the effect of MPSSS on T cell proliferation inhibition by prostate CAFs, we used Western blotting to detect the effect of MPSSS on α-SMA expression in prostate CAFs. The results showed that the expression of α-SMA decreased after incubation with different concentrations of MPSSS (0–0.5 mg/ml) for 24 h, and a dose-dependent relationship existed. Though detecting only one of the proteins highly expressed in CAFs is not enough to explain how MPSSS impacts the immunosuppressive function of CAFs, the results indicate that MPSSS could change the activation state of CAFs; this is a possible mechanism by which MPSSS reversed the immunosuppressive effect of CAFs (Figure 6).

## Discussion

Prostate cancer is a kind of cancer with high prevalence and mortality. At present, researchers are looking for suitable methods to effectively treat prostate cancer [16]. By targeting prostate CAFs, our study found that MPSSS does not directly inhibit the growth of prostate CAFs but can change the immunosuppressive function of CAFs, down-regulate the expression of α-SMA, and thus reduce their activity. Natural components extracted from traditional Chinese medicinal compounds have been demonstrated to have certain therapeutic effects in various diseases, and many natural plant extracts, such as paclitaxel and lentinan, display antitumor effects after isolation and purification [9,17]. In previous studies, we introduced a new polysaccharide from *L. sedodes*, MPSSS, and demonstrated *in vitro* that MPSSS can reduce the immunosuppressive function of MDSCs and can inhibit tumor growth [11].

MPSSS does not inhibit the growth of prostate CAFs but modifies CAFs by changing their function in the treatment of tumors and effectively reduces the side effects. In the tumor microenvironment, fibroblasts are located at the junction of the tumor endothelium and peripheral tissues, which hinder the response of the immune system against tumors. Fibroblasts from malignant tumors also express proteins that are inhibited in normal tissue fibroblasts [18], especially α-SMA and FAP. CAFs highly express these proteins and inhibit the proliferation of immune cells and also inhibit the antitumor immune response [4,19]. For this reason, we assume that preventing the inhibition of the immune response by CAFs may be an effective and feasible method to treat tumors. After incubation with MPSSS, the inhibitory effect of prostate CAF supernatant on T cell proliferation was reduced, indicating that MPSSS could alter the secretion of some immunosuppressive or immunostimulating factors by prostate CAFs and could affect the components of the CAF supernatant. Further verification needs to be performed by detecting the changes in the cytokine levels in the supernatant of prostate CAFs. The inhibition of T cell proliferation was caused by CAFs through the indirect action of cytokines, and this inhibition may also be through direct celluar interactions. After the co-culture of MPSSS-treated prostate CAFs with splenocytes, the proliferation of T cells was inhibited, which demonstrated the possibility of direct contact between CAFs and T cells; however the specific mechanism could not be determined and remains to be further studied.

It has been proven that MPSSS impairs the immunosuppressive function of MDSCs through the MyD88-dependent NF-κB pathway [11]. Therefore, we speculated that MPSSS affected the function of prostate CAFs in a similar way. Western blot analysis revealed that MPSSS changed the protein expression at the key nodes of the TLR4-NF-κB signaling pathway, indicating that the TLR4-NF-κB pathway is closely related to the role of MPSSS. As an important protein in the innate immune response, TLR4 is expressed in many important tumor cells and immune cells, which is also involved in the antitumor functions of some traditional Chinese medicine [20,21]. TLR4 recruits MyD88 to its TIR domain, and then MyD88 mediates the binding of downstream TRAF6 to the TAB-1/TAK1/TAB2 membrane complex, resulting in the phosphorylation of TAK1 and the release of the complex from the membrane. Activated TAK1 phosphorylates the IKK complex, leading to the phosphorylation and activation of p65. Then, NF-κB molecules are released and translocated to the nucleus, where they bind to target genes to promote the expression of inflammatory factors [14]. Thus, we examined the expression of seven proteins related to the TLR4-NF-κB pathway. The key protein molecules (pIKK, p-TAK, TAK, TRAF6, p-P65 P65, and MyD88) were found to activate the MyD88-dependent TLR4-MyD88 pathway. The pretreatment of prostate CAFs with the TLR4 antagonist CIL-095 abrogated the change in protein expression caused by MPSSS, which confirmed the importance of TLR4 as an MPSSS receptor. However, the final effects of activating this pathway remain to be studied. NF-κB activation may cause prostate CAFs to secrete factors that promote their immune response to improve their immunosuppressive function, but the specific mechanism needs to be further determined.

We also demonstrated that MPSSS could down-regulate the expression of α-SMA in prostate CAFs. The α-SMA is a marker of activated fibroblasts and is mostly expressed in CAFs [4]. It has been suggested that MPSSS has a certain effect on changing CAF properties; perhaps MPSSS can promote the transformation of CAFs into NAFs. However, how MPSSS changes the properties of CAFs needs to be further studied.

## Abbreviations

α-SMA: α*-s*mooth muscle actin
APC: allophycocyanin
BCA: bicinchoninic acid
CAF: cancer-associated fibroblast
CFSE: carboxyfluorescein succinimidyl amino ester
CXCL: chemokine (C-X-C motif) ligand
FBS: fetal bovine serum
GADPH: glyceraldehyde-3-phosphate dehydrogenase
IKK: IkappaB kinase
IL: Interleukin
LPS: lipopolysaccharide
MDSC: myeloid-derived suppressor cell
MTT: 3-[4,5-dimethylthiazol-2-yl]-2,5-diphenyltetrazolium bromide
MyD88: myeloid differentiation factor 88
PBS-T: phosphate buffered saline-tween
PE: phycoerythrin
TAK: TGF beta-activated kinase
TLR: Toll-like receptor
TRAF: TNF receptor-associated factor
